# The role of declining therapy volumes in skilled nursing facility outcomes: a mediation analysis

**DOI:** 10.1093/haschl/qxag029

**Published:** 2026-02-07

**Authors:** Rachel A Prusynski, Andrew Humbert, Harsha Amaravadi, Robert E Burke, Debra Saliba, Natalie E Leland, Janet Freburger, Tracy M Mroz

**Affiliations:** University of Washington Department of Rehabilitation Medicine, Seattle, WA 98195, United States; University of Washington Department of Health Systems and Population Health, Seattle, WA 98195, United States; University of Washington Department of Rehabilitation Medicine, Seattle, WA 98195, United States; University of Washington Department of Health Systems and Population Health, Seattle, WA 98195, United States; University of Pennsylvania Perelman School of Medicine, Philadelphia, PA 19104, United States; Corporal Michael J. Crescenz Veterans Affairs Medical Center, Philadelphia, PA 19104, United States; Borun Center, Division of Geriatrics, University of California Los Angeles, Los Angeles, CA 90024, United States; Geriatric Research Education and Clinical Center, Greater Los Angeles Veterans Affairs Medical Center, Los Angeles, CA 90073, United States; RAND Health, Santa Monica, CA 90401, United States; University of Pittsburgh Department of Occupational Therapy, Pittsburgh, PA 15219, United States; University of Pittsburgh Department of Physical Therapy, Pittsburgh, PA 15219, United States; University of Washington Department of Rehabilitation Medicine, Seattle, WA 98195, United States; University of Washington Department of Health Systems and Population Health, Seattle, WA 98195, United States

**Keywords:** skilled nursing facilities, mediation analysis; patient readmission, patient discharge, physical therapy, occupational therapy, speech language pathology, Medicare

## Abstract

**Introduction:**

Significant declines in therapy provision in skilled nursing facilities (SNFs) followed the 2019 implementation of Medicare's Patient-Driven Payment Model (PDPM) and the onset of the COVID-19 pandemic, raising concerns about effects on patient outcomes.

**Methods:**

Using Medicare fee-for-service claims and SNF assessment data from January 2018 through September 2021, we analyzed 3.5 million post-hospital SNF stays to assess whether changes in therapy volumes mediated changes in successful community discharge and 30-day hospital readmissions.

**Results:**

Average total physical, occupational, and speech therapy minutes per day declined from 122.2 before PDPM to 96.5 immediately after implementation and to 87.7 during the pandemic. Adjusted probabilities of successful community discharge rose modestly after PDPM but fell during COVID-19, while readmissions declined initially and then increased. Mediation analyses showed that reductions in therapy volumes were strongly associated with the declines in community discharge and increases in readmissions. These findings persisted for patients with dementia and moderate levels of functional impairment at admission; declining therapy volumes were associated with the observed worsening of discharge outcomes after PDPM implementation and during the pandemic.

**Conclusions:**

Results highlight therapy provision as a key modifiable policy target for improving post-acute outcomes and reducing rehospitalizations among older adults in SNFs.

## Introduction

For the 1.6 million Medicare fee-for-service (FFS) beneficiaries admitted to skilled nursing facilities (SNFs) after hospitalization annually,^1^ two outcomes are highly prioritized as markers of a successful post-hospital SNF stay: discharge to the community and avoiding rehospitalization.^1-3^ These outcomes are crucial targets for patients and caregivers as well as SNFs, payers, and health systems that work towards improving quality of care and reducing overall healthcare costs.^1,4-8^ Care provided during post-hospital SNF stays includes nursing and rehabilitation (ie, physical, occupational, and speech therapy) services. Therapy services have the potential to help SNFs achieve successful community discharge and avoid hospital readmissions by helping patients improve functional mobility, safety, and independence with activities of daily living.^9^ Indeed, impaired functional status has been a strong predictor of increased risk of readmissions across multiple studies,^4,10-12^ and higher volumes of therapy services (ie, more minutes of therapy) during SNF stays have been associated with functional improvement and improved community discharge.^9,13^ However, the question of whether more therapy should be encouraged, and for which patients, remains unclear.^14^

Therapy volumes in SNFs have declined substantially in recent years due to two major events—SNF payment policy reform and the COVID-19 pandemic.^15^ In October 2019, in response to sharply increasing therapy volumes during the prior twenty years,^16^ CMS implemented a new SNF payment policy for FFS stays, the Patient-Driven Payment Model (PDPM).^17^ PDPM intended to reduce potentially low-value therapy provision by removing the financial incentives for SNFs to provide high-volume therapy services at specific levels just above payment thresholds.^14^ The COVID-19 pandemic, which began shortly after PDPM implementation, was also highly disruptive for SNF admissions, staffing, and other operations.^18-20^ Our prior work found that, together, these two events led to a reduction of nearly 24% in average minutes per day of therapy provided to FFS patients in SNFs.^15^ However, the impacts of declining therapy volumes on patient outcomes during this period are unclear, given challenges in disentangling the independent effects of PDPM and the pandemic.^21^ Early research evaluating PDPM reports minimal changes in patient outcomes immediately after implementation,^22-24^ however, these studies were all limited to the 5 months between PDPM implementation and the pandemic period. More recently, the Medicare Payment Advisory Commission reported small declines in successful community discharge and fluctuating readmission rates at SNFs from 2019 to 2021.^21^ However, these descriptive analyses do not take into account changes in patient characteristics, despite evidence that SNFs admitted increasingly complex patients after PDPM and during the pandemic.^25,26^ Additionally, to our knowledge, no work has specifically examined how declining therapy volumes, resulting from PDPM and the pandemic, impacted community discharge and readmission outcomes. Because therapy volumes can be modified by SNFs through operational staffing changes, understanding the relationships between therapy volumes and patient outcomes is essential for guiding SNFs in implementing patient-centered care delivery that can maximize positive outcomes.

To fill this gap, we conducted a mediation analysis to estimate the extent to which the observed declining therapy volumes mediated the association between PDPM implementation and the COVID-19 pandemic and rates of community discharge and readmission.^27,28^ By conceptualizing therapy volume as a mediator on the causal pathway between our exposures (1) PDPM implementation and (2) the pandemic and our outcomes, we attempt to isolate the indirect effects of declining therapy volumes in the pathway to help inform guidance on therapy provision in SNFs.

## Methods

This study was approved by the University of Washington institutional review board, which waived the requirement for informed consent. The study followed the Strengthening the Reporting of Observational Studies in Epidemiology (STROBE) reporting guidelines.

### Cohort creation

We used 100% Medicare Provider Analysis and Review (MedPAR) hospital and SNF claims from January 2018 through September 2021 to identify FFS hospital stays that had a subsequent SNF stay within three days of hospital discharge. We only included beneficiaries whose continuous enrollment in FFS Medicare could be verified using Master Beneficiary Summary Files (MBSF) for three consecutive months after hospital discharge. We then merged MedPAR claims with the Minimum Data Set (MDS) 3.0 to identify SNF stays with complete admission and discharge MDS assessments. Discharge assessments are only available for patients who do not die during the SNF stay, so those patients were excluded. The flowsheet for cohort creation is in Appendix Figure S1.

### Outcomes

Our outcomes were successful discharge to the community and 30-day hospital readmissions after SNF discharge. Consistent with CMS methods,^29^ we used MDS discharge assessments combined with MedPAR hospital claims and MBSF data to create each outcome. Successful community discharge occurred when a patient discharged from the SNF to a community setting (eg, home with or without home health services) with no subsequent acute care, SNF, or long-term hospital admissions or death within 30 days of discharge. Hospital readmissions occurred if we identified any acute or long-term care hospital admissions within 30 days of SNF discharge, regardless of whether the initial discharge setting after the SNF stay was to a community setting or another institutional setting.

### Exposures

Our exposures were time-based indicators for the month of implementation of PDPM in October 2019 and the onset of the COVID-19 pandemic in March 2020.^30^ Each SNF stay was assigned to one of three study time periods based on the month of admission: period 1: Before PDPM implementation (January 2018–September 2019), period 2: After PDPM implementation, before COVID-19 onset (October 2019–February 2020), and period 3: After COVID-19 onset (March 2020–September 2021).

### Confounders

We included many patient and SNF-level covariates from MedPAR, MBSF, MDS, and CMS public SNF data sources (ie, LTCFocus, Provider of Services Files, Payroll Based Journal, Care Compare),^31-33^ as well as daily county-level COVID case rates averaged across the dates of the SNF stay.^34^ Patient-level covariates included demographics (ie, age, sex, self-reported race and ethnicity, dual eligibility for Medicare and Medicaid, marital status, and rurality of the patient's home ZIP Code). For medical severity, we included indicators for hospital surgical procedures and intensive care unit stays, an Elixhauser comorbidity index calculated from diagnoses on the hospital claim, and diagnoses of depression or Alzheimer's Disease or related dementias from MBSF chronic conditions files.^35^ From MDS assessments, we included whether the patient required an interpreter, vision impairments, receipt of hospice care, chemotherapy, hemodialysis, or a ventilator, indicators for pain affecting sleep or activity, delirium, pressure sores, incontinence, agitated behaviors,^36^ recent falls, and a combined functional status score for seven activities of daily living on admission.^37^

We included time-varying facility-level variables from SNF public files such as facility ownership status and monthly average patient census and hours of staffing for registered nurses, licensed practical nurses, and certified nurse assistants.^31^ We also included annual five-star quality of care ratings, chain status, urban or rural county, freestanding versus in-hospital location, and payor mix (ie, percent of Medicare and Medicaid patients).^32,33^

### Mediator

Our mediator was therapy volume, measured as minutes of therapy per day of therapy during the SNF stay. For all stays, we summed minutes of speech, occupational, and physical therapy and days of therapy for each discipline from MDS assessments to calculate therapy volume. It should be noted that PDPM changed the cadence of MDS reporting by removing required interim assessments (eg, 14- and 30-day assessments). As a result, slightly different sets of assessments were used to create therapy volume measures after October 2019. New items were also added to MDS discharge assessments that included all therapy minutes and days of therapy across the full SNF stay after PDPM.^38^

Thus for period 1, in the absence of the total minutes item for the full stay, we summed total minutes from each individual admission, interim, and discharge assessment and divided by the sum of all days of therapy from each assessment's lookback period to create minutes of therapy per day of therapy. For periods 2 and 3, in the absence of interim assessments, we used the new summary items on the MDS discharge assessment for total therapy minutes and total days of therapy during the whole stay. Therapy volume during period 1 would not include minutes or days of therapy not captured during an interim assessment lookback period for longer stays (eg, days 15-21 of the stay would not be captured on the 14- and 30-day interim assessments or a discharge assessment for a stay over 36 days long). Yet, this concern applies to a minority of longer post-acute SNF stays considering the average stay in our cohort was about 25 days long. This method was used in prior studies to calculate SNF therapy volumes which found similar declines in therapy after PDPM.^15,22,39^

### Statistical analysis

Multivariable linear regression was used to estimate the individual effects of two exposures: PDPM implementation and the onset of the COVID-19 pandemic, on each outcome (ie, the probability of successful community discharge and the probability of 30-day hospital readmission), explicitly excluding therapy volumes in order to capture the total effects of the exposures.^27^ We estimated linear probability models to facilitate direct interpretation of effects on the probability scale and to support decomposition of total effects into direct and indirect components in our mediation analysis. Prior work demonstrates that linear models provide close approximations to average marginal effects from logistic models when samples sizes are large and are particularly useful when fixed effects and clustered standard errors are required.^40^

We estimated two total effects models—one with community discharge as the outcome and one with hospital readmission as the outcome. In both models, we included time-based indicators for PDPM implementation (October 2019) and COVID-19 onset (March 2020) as separate exposures. Each indicator's coefficient separately estimates the change in the outcome associated with that exposure, relative to the pre-PDPM baseline period, holding the other indicator constant. To account for seasonality, we added fixed effects for calendar month. We included all time-varying confounders at the patient and facility levels described above and clustered standard errors at the SNF and beneficiary-levels to account for repeated measures. Finally, we included fixed effects for facility to capture residual confounding induced by other changes at the facility level after PDPM implementation or during the pandemic.

After estimating total effects models, we conducted a mediation analysis to determine the indirect effects PDPM implementation and COVID-19 had on each outcome through their associations with declining therapy volumes (the mediator).^27,41^ The directed acyclic graph that depicts the hypothesized causal pathway between exposures and outcomes and the role of the mediator is included in Appendix Figure S2, which also includes model equations (Appendix Table S1). For the mediation analysis, we estimated two additional linear models (Models 1 and 2 in Appendix Table S1) for each outcome with the same covariates as the total effects models described above. First, we used a model with PDPM implementation, COVID-19 onset, and therapy volume as predictors of each patient outcome, to estimate the association of decreasing therapy volumes (as well as the direct effect of PDPM and COVID-19) on the patient outcomes, referred to as direct effects. Direct effects can be interpreted as the hypothetical change in outcomes had the exposures not reduced therapy volumes. Next, we estimated associations between PDPM implementation and COVID-19 and therapy volumes to estimate how therapy volumes changed across the time periods. The estimated effects of (a) PDPM implementation and COVID-19 on therapy volumes and (b) therapy volumes on the patient outcomes were then multiplied to determine the indirect effect of PDPM implementation and COVID-19 onset mediated through declining therapy volumes. We calculated standard errors for indirect effects using the multivariate delta method, and 95% confidence intervals using normal approximation.^42^ Consistent with prior literature,^28^ we intentionally omitted length of stay from all models because changes in length of stay would be considered an additional mediator between the exposures and outcomes, and conditioning on other mediators can inadvertently induce bias.^27^

We conducted a series of sensitivity analyses to address potential differences in the outcomes, confounders, and the mediator across our study period, particularly during the COVID-19 pandemic. First, we divided the pandemic period into early and late phases using December 2020 as the cut-off, consistent with prior literature^18^ because this was when COVID-19 vaccines became available and when we also noticed a change in trends for the mediator, although the outcomes fluctuated during the pandemic period with no obvious trends (See Appendix Figure S3).

Then, we conducted two stratified analyses to examine heterogeneity in the effects of PDPM implementation, COVID-19 onset, and the mediating effect of declining therapy volumes on different subsets of patients based on clinical characteristics. First, we stratified by dementia diagnosis because PDPM led to declines in SNF admissions for patients with dementia and dementia diagnosis may influence patients' ability to participate in high-volume therapy during post-acute SNF stays.^23,26^ Second, we divided SNF stays based on quartiles of the functional status score at admission. Patients with different levels of functional impairment may have different abilities to participate in therapy and be differentially impacted by declines in therapy volumes that occurred after PDPM implementation and the pandemic, and there is evidence that declines in therapy mediated worse functional outcomes for patients in SNFs.^28^ All analyses were conducted in R Version 4.5.0 between June and December 2025.

## Results

**Figure 1.**
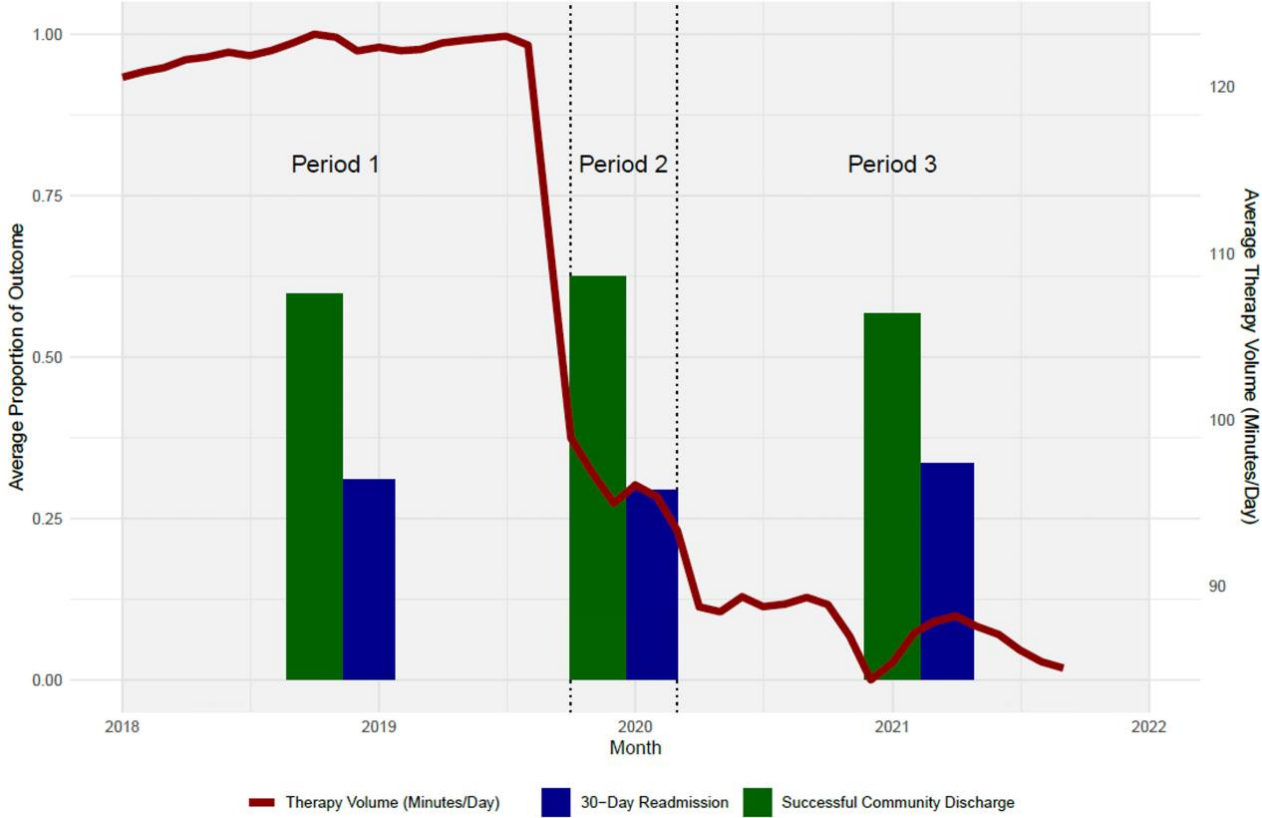
Unadjusted average therapy volumes and patient outcomes over time among 3 534 928 skilled nursing facility (SNF) stays. Bars reflect the average proportion of SNF stays with successful community discharge and 30-day readmissions by time period. The red line shows the monthly average therapy volume, or total minutes of physical, occupational, and speech therapy per day of therapy during the SNF stay. Period 1: Before Patient-Driven Payment Model (PDPM) implementation (January 2018 through September 2019); period 2: After PDPM Implementation, Before COVID-19 onset (October 2019 through February 2020); period 3: After COVID-19 onset (March 2020 through September 2021). *Source:* Authors' analysis of 2018-2021 Medicare Provider Analysis and Review, Master Beneficiary Summary Files, Minimum Data Set 3.0, and publicly available files from the Centers for Medicare and Medicaid Services.

We had complete admission and discharge data for 3 534 928 post-acute FFS SNF stays between January 2018 and September 2021 (Appendix Figure S1). Among those stays, unadjusted average therapy volumes declined from 122.2 minutes of therapy per day in period 1 to 96.5 minutes per day in period 2, and therapy volumes declined further to 87.7 average minutes per day in period 3 (Figure 1). For unadjusted outcomes, in period 1, 58.5% of stays ended in a successful community discharge, which increased to 61.3% in period 2 but decreased to 55.0% in period 3. In period 1, 30.9% of stays resulted in a hospital readmission within 30 days, which declined to 29.4% in period 1 and then increased to 33.5% in period 3. Descriptive statistics for outcomes, therapy volumes, and all patient and facility-level covariates across the three study periods are in Appendix Table S2.

### Effects of PDPM implementation

**Figure 2.**
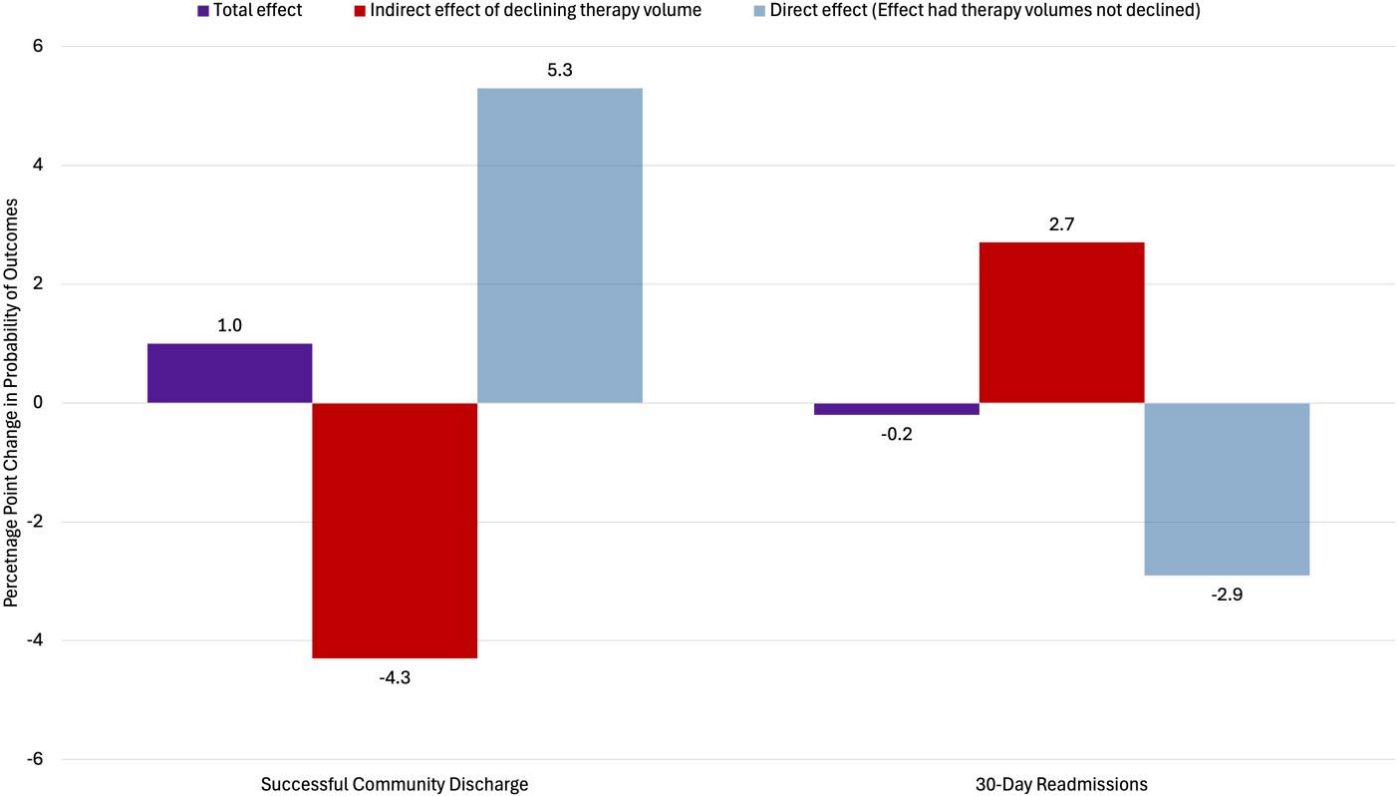
Adjusted effects of Patient-Driven Payment Model (PDPM) implementation on patient outcomes with mediation analysis estimates of indirect effects of declining therapy volumes for 3 534 928 skilled nursing facility (SNF) stays from January 2018 through September 2021. Purple bars show the total effect estimates of PDPM implementation on the outcomes. Red bars show the mediation analysis estimates of the indirect effects of declining therapy volumes on the outcomes. Light blue bars show the direct effect, or hypothetical effect of PDPM on each outcome in the absence of the mediating indirect effect of declining therapy volumes. *Source:* Authors' analysis of 2018-2021 Medicare Provider Analysis and Review, Master Beneficiary Summary Files, Minimum Data Set 3.0, and publicly available files from the Centers for Medicare and Medicaid Services.

**Figure 3.**
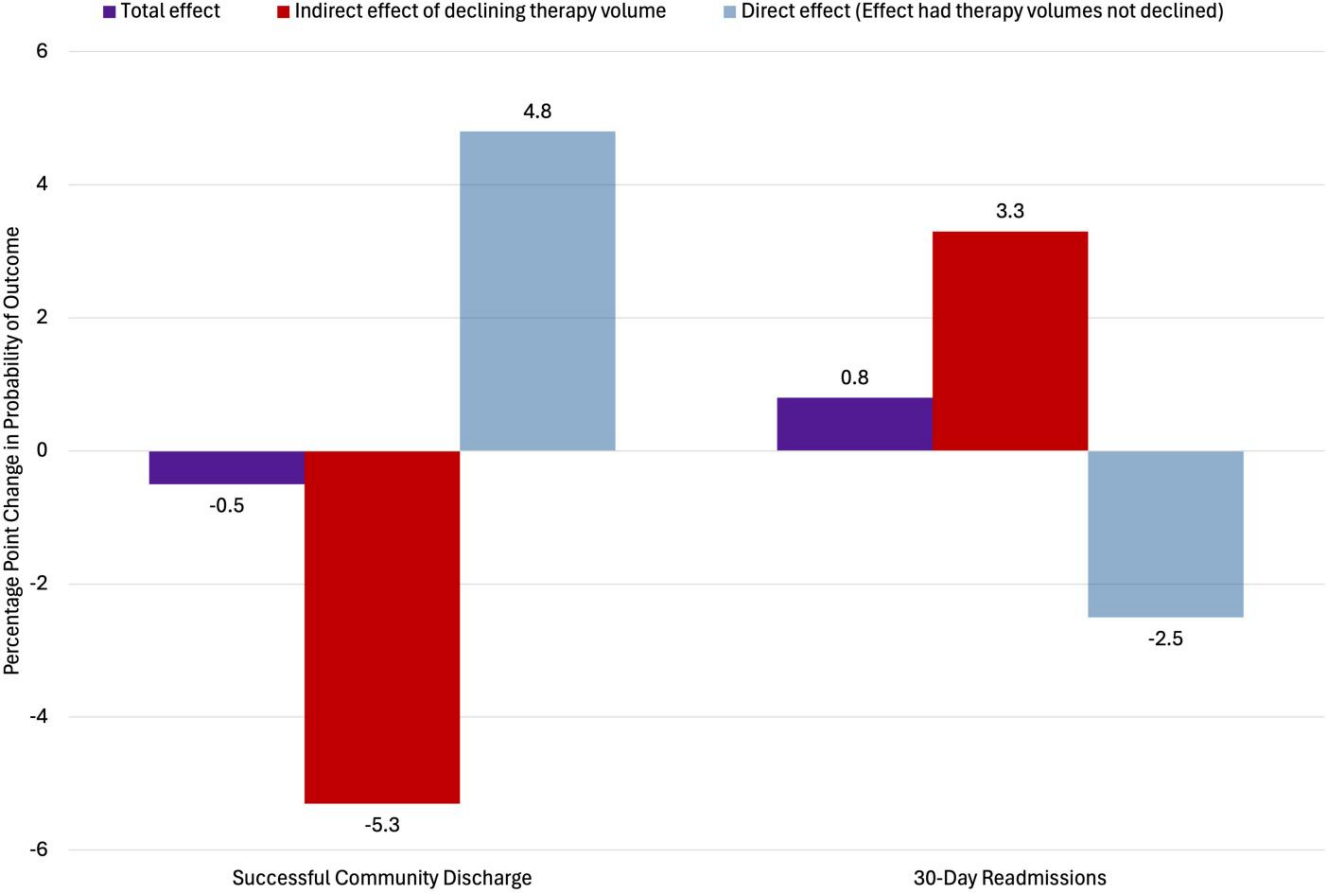
Adjusted effects of COVID-19 onset on patient outcomes with mediation analysis estimates of indirect effects of declining therapy volumes for 3 534 928 skilled nursing facility (SNF) stays from January 2018 through September 2021. Purple bars show the total effect estimates of COVID-19 onset on the outcomes. Red bars show the mediation analysis estimates of the indirect effects of declining therapy volumes on the outcomes. Light blue bars show the direct effect, or hypothetical effect of COVID-19 on each outcome in the absence of the mediating indirect effect of declining therapy volumes. *Source:* Authors' analysis of 2018-2021 Medicare Provider Analysis and Review, Master Beneficiary Summary Files, Minimum Data Set 3.0, and publicly available files from the Centers for Medicare and Medicaid Services.

Estimates of total, direct, and indirect effects of PDPM implementation are included in Table 1. In adjusted analyses, the total effect of PDPM implementation was a 1.0% point (pp) increase (95% CI 0.8, 1.1) in the probability of successful community discharge compared with the period 1 baseline. As seen in Figure 2, the mediation analysis estimated that the decline in therapy volumes after PDPM implementation had a negative indirect effect of −4.3pp (95% CI −4.3, −4.2) on the probability of successful community discharge. For hospital readmissions, the total effect of PDPM implementation was a 0.2pp reduction (95% CI −0.4, −0.05) compared with baseline. Mediation analysis (Figure 3) estimated that the decline in therapy volumes after PDPM implementation had a positive indirect effect of 2.7pp (95% CI 2.6, 2.8) on the probability of 30-day hospital readmissions.

**Table 1.** Effects of Patient-Driven Payment Model (PDPM) implementation and COVID-19 onset on patient outcomes compared to baseline period (January 2018 through September 2019) with mediation analysis estimates of indirect effects of declining therapy volumes for 3 534 928 skilled nursing facility stays from January 2018 through September 2021

	Total effects (95% CI)	Direct effects (95% CI)	Indirect effects (95% CI)
***Percentage point change in probability following pdpm implementation***			
Successful Community Discharge	1.0 (0.8, 1.1)	5.3 (5.1, 5.5)	−4.3 (−4.4, −4.2)
30-Day Hospital Readmissions	−0.2 (−0.4, −0.05)	−2.9 (−3.1, −2.7)	2.7 (2.6, 2.8)
***Percentage point change in probability following COVID-19 onset***			
Successful Community Discharge	−0.5 (−0.7, −0.3)	4.8 (4.6, 5.0)	−5.3 (−5.4, −5.2)
30-Day Hospital Readmissions	0.8 (0.6, 1.0)	−2.5 (−2.7, −2.3)	3.3 (3.3, 3.4)

Effects are estimated from multivariable linear regression models with facility and calendar month fixed effects and adjusted for time-varying beneficiary and facility-level characteristics. Standard errors were clustered at the beneficiary and facility level to account for repeated measures. Total effects reflect estimated changes in each outcome after PDPM implementation and COVID-19 onset compared to baseline and accounting for declining therapy volumes. Direct effects are adjusted estimates of PDPM and COVID-19 onset in the absence of the mediator of declining therapy volumes. Indirect effects are results of the mediation analysis, estimating the indirect effects of changes in therapy volumes (ie, minutes of therapy per day) on the total effects. *Source*: Authors' analysis of 2018-2021 Medicare Provider Analysis and Review, Master Beneficiary Summary Files, Minimum Data Set 3.0, and publicly available files from the Centers for Medicare and Medicaid Services.

### Effects of COVID-19 onset

The total effect of COVID-19 pandemic onset was a 0.5pp reduction (95% CI −0.7, −0.3) in the probability of successful community discharge compared with period 1 baseline. As seen in Figure 3, after COVID-19 onset, declining therapy volumes had a negative indirect effect of −5.3pp (95% CI −5.4, −5.2) on the probability of successful community discharge. For hospital readmissions, the total effect of COVID-19 pandemic onset was a 0.8pp increase (95% CI 0.6, 1.0) compared with baseline. Declining therapy volumes had a positive indirect effect of 3.3pp (95% CI 3.3, 3.4) on the probability of 30-day hospital readmissions after pandemic onset.

### Sensitivity analyses

Results for total, direct, and indirect effects of PDPM and the onset of COVID-19 in March 2020 were similar to primary analyses when respecifying period 3 into early and late COVID-19 periods (Appendix Table S3). Compared to period 1 baseline, the total effect of the onset of the late COVID-19 period in December 2020 was a −2.0pp change in the probability of successful community discharge (95% CI −2.5, −1.6) and the indirect effect of declining therapy volumes was a 5.0pp reduction (95% CI −5.2, −4.9). The total effect of late COVID-19 period onset was a 1.9pp increase in the probability of hospital readmissions (95% CI 1.5, 2.4) and the indirect effect of declining therapy volumes was an increase of 3.2pp (95% CI 3.1, 3.3).

Results of stratified analyses by dementia diagnosis are in Appendix Table S4. Directions and effects sizes of the total, direct, and indirect effects were largely similar to primary analyses, with the exception of no statistically significant total effect of PDPM implementation or COVID-19 onset on 30-day readmissions for beneficiaries without dementia. Beneficiaries with dementia had larger effect sizes for the indirect effects of declining therapy volumes on both outcomes during both time periods compared with those without dementia.

Results of stratified analyses by level of function (low, moderate, high) based on functional score at admission are in Appendix Table S5. Beneficiaries with low function experienced the largest total positive effects of PDPM implementation on both outcomes, consistent with smaller indirect effects of declining therapy volumes relative to those with moderate levels of function. Patients with high function also experienced smaller indirect effects of declining therapy volumes compared with those with moderate function, consistent with smaller total effects of PDPM implementation on successful community discharge and no statistically significant total effect of PDPM implementation or COVID-19 onset on 30-day readmissions for beneficiaries with high function at admission.

## Discussion

In this analysis of the mediating effects of therapy volume on the effects of PDPM implementation and the COVID-19 pandemic, we found that declining therapy volumes were negatively associated with successful community discharge and positively associated with increased risk of 30-day hospital readmissions after both PDPM implementation and during the pandemic. These findings are robust to the use of different pandemic time frame indicators and suggest that, had therapy volumes not declined to such an extent, FFS beneficiaries may have experienced more successful community discharges and fewer rehospitalizations after SNF payment reform and the pandemic.

The direct effect estimates from the mediation analysis reflect the hypothetical change in outcomes after PDPM and the COVID-19 pandemic had the exposures not reduced therapy volumes. Specifically, our results suggest that, in the absence of declining therapy volumes, successful community discharge rates could have been about 5pp higher—and rehospitalization rates could have been about 3pp lower—during both periods. However, these relatively large direct effects may also reflect unmeasured confounding, especially if patient case mix and other care delivery practices in SNFs changed in ways that could not be operationalized in administrative data or captured through our use of facility fixed effects.

The total effect estimates, which do not account for differences in therapy volumes, suggest PDPM implementation was slightly beneficial for both outcomes; specifically, community discharge rates increased by 1.0pp and readmissions decreased by 0.2pp. These small positive changes are consistent with prior literature and CMS reports stating that outcomes were improving prior to PDPM implementation and were relatively stable immediately after payment reform.^21-23^ These small improvements may also be a continuation of positive trends in outcomes that began in the mid-2010s when Congress passed multiple measures aimed at improving quality of SNF care.^21,43,44^ Our stratified sensitivity analyses shed additional light on the mechanism for these small positive PDPM effects, which were larger for patients with dementia and those with the lowest function at admission. While patients with dementia experienced large negative indirect effects of declining therapy volumes, it is possible that other aspects of PDPM's emphasis on cognitive and functional status when calculating reimbursement rates specific to nursing and non-therapy ancillary needs were helpful in incentivizing SNFs to provide more patient-centered care outside of therapy provision. Indeed, while evidence suggests fewer patients with dementia were admitted to SNFs after PDPM, dementia admissions declined more in low-quality facilities,^25,26^ such that the remaining patients with dementia were admitted to medium and high quality facilities where they may have received better nursing care. Patients across function quartiles all experienced indirect effects of declining therapy volumes associated with worsening for both outcomes after PDPM and the pandemic, but the effect sizes were largest for those with moderate levels of functional impairment. This may suggest that patients with moderate functional impairment benefit most from higher therapy volumes—and therefore experience the worst relative impacts of therapy volume declines—during their SNF stay.^28^

Once the pandemic began, adjusted successful community discharge rates declined by an estimated 0.5pp and 30-day hospital readmissions increased by 0.8pp. In addition to declining therapy volumes which we explicitly account for in the mediation analysis but are not adjusted for in the total effect estimates, these worse outcomes may be related to other disruptions in care delivery, staffing, patient case mix, patient and staff infections, and COVID-19 mitigation efforts.^18,20,45,46^

Our mediation analysis results have particular salience as the SNF Value-Based Purchasing (VBP) Program instituted in 2018 by CMS creates a mandatory pay-for-performance program for SNFs to reduce rehospitalization rates and, in the future, improve community discharge rates.^47^ While the SNF VBP program was not successful in reducing readmissions within 30 days of hospital discharge, our results suggest SNFs may improve performance by striking a balance between appropriately intensifying therapy delivery without reverting to threshold-based therapy provision that was characteristic of the Pre-PDPM era.^14,48^ A cost-benefit analysis in future work could help identify to what degree investments in therapy staffing could translate into larger bonuses in SNF VBP payments. This is particularly important for SNFs that are low-performing at baseline, since these SNFs may ultimately have fewer resources available to make staffing investments.^48^

To increase therapy volumes, SNFs may also consider lower-cost practices for therapy provision, such as including multiple patients in each therapy session, or utilizing more lower-paid therapist assistants in lieu of higher-paid therapists.^49,50^ While our analysis included all therapy minutes regardless of number of patients in each session or provider type, some research conducted prior to PDPM implementation suggests these lower-cost therapy practices may be positive for patient outcomes as long as they do not dominate therapy provision.^51,52^ Further research, however, is needed to understand the impacts of lower-cost therapy practices in the context of PDPM, under which overall therapy volumes are lower.

### Limitations

We acknowledge multiple limitations to this study. While mediation analysis assumes the models are fully specified, the observational design and use of administrative data cannot eliminate the potential for unmeasured confounding. While we attempted to adjust for a comprehensive set of patient and time-varying facility factors alongside facility fixed effects, ultimately, results should be interpreted as associations only. As noted above, we did not account for type of therapy provider (ie, assistant vs. therapist), number of patients in a therapy session, or the quality of therapy care, which may have an impact on the outcomes we examined. However, these aspects of care delivery may also be considered alternative mediators to declining therapy volumes as they could be on the causal pathway between our exposures and outcomes, so including them could induce additional bias.^27^ Observations without facility-level data in public files were also excluded, which may mean results are not as generalizable to smaller SNFs that are more likely to have incomplete data.^53^ We also did not include Medicare Advantage data, so results only apply to the FFS population, though other work has suggested spillover effects of PDPM on the growing Medicare Advantage population in SNFs.^54^ We examined all planned and unplanned readmissions within 30 days of SNF discharge because we anticipated all readmissions could have been impacted by declining therapy volumes, but we acknowledge that CMS' 30-day readmission measure under the SNF VBP uses an algorithm to exclude planned readmissions. Additionally, CMS' measure uses hospital discharge as the starting date for 30-day readmissions compared with our use of SNF discharge as the starting date, so our results should not be used to predict changes in payment adjustments under the SNF VBP.^47^

## Conclusions

In this study of 3.5 million SNF admissions among FFS Medicare beneficiaries between 2018 and 2021, we found that successful community discharge and 30-day hospital readmission outcomes improved slightly after Medicare payment reforms but became slightly worse during the COVID-19 pandemic. However, declining therapy volumes may have contributed to worsening of both outcomes across both time periods, suggesting that, had therapy volumes not declined to such an extent, patients receiving care in SNFs may have experienced even more successful discharges to the community and fewer hospital readmissions. Policies directly incentivizing appropriate therapy volumes, especially for patients with dementia and those with moderate levels of functional impairment at admission, could lead to better outcomes. Finally, SNFs seeking to improve outcomes and potentially earn higher payments under the SNF VBP may consider increasing therapy volumes.

## Supplementary Material

qxag029_Supplementary_Data
