# Supplementary material for: The role of declining therapy volumes in skilled nursing facility outcomes: a mediation analysis
Source: Health Aff Sch. 2026 Feb 7;4(2):qxag029. doi: 10.1093/haschl/qxag029 (PMC12927502; doi:10.1093/haschl/qxag029)
Supplement: qxag029_Supplementary_Data [file qxag029_supplementary_data.zip › dc_readmission_appendix_revision (1).docx]

**Appendix**

**The role of declining therapy volumes in skilled nursing facility outcomes: a mediation analysis**


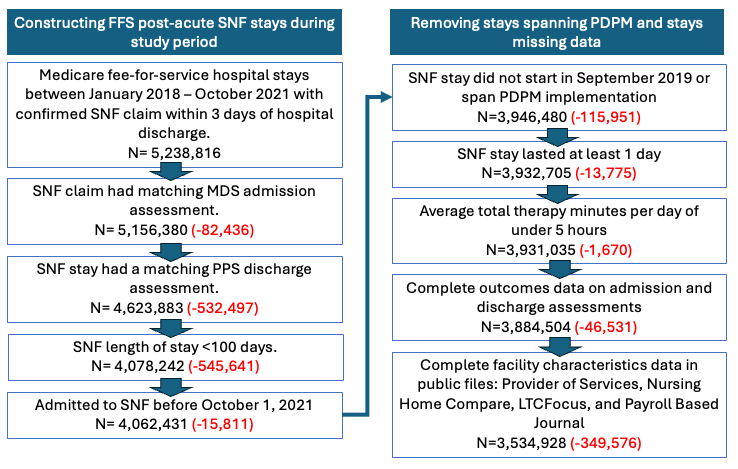


**Appendix Figure 1**. Flowsheet for cohort creation.

**Notes:** Process for creating fee-for-service (FFS) skilled nursing facility (SNF) stays by merging hospital claims with Minimum Data Set (MDS) 3.0 assessments. PPS – prospective payment system. PDPM – Patient-Driven Payment Model.

| **Appendix Table 1. Models and effect estimates in mediation analysis** | |
| --- | --- |
| **Model** | **Description and Equation** |
| Model 0 | Total effect of Exposure(X) $\to$ Outcome(Y)   - Y = γ₀ + γ₁X + ε₃ |
| Model 1 | Effect of Exposure (X) and Mediator (M) $\to$ Outcome(Y)   - Y = β₀ + β₁X + β₂M + ε₂ |
| Model 2 | Effect of Exposure(X) $\to$ Mediator(M)   - M = α₀ + α₁X + ε₁ |
| Indirect Effect (IE) | Multiply β₂ (from model 1) by α₁ (from model 2)   - α₁ × β₂ |
| Direct Effect (DE) | β₁ from Model 1 |
| Total Effect (TE) | - γ₁ (from Model 0) = β₁ + (α₁ × β₂), or TE = DE + IE |

**Appendix Figure 2.** Directed acyclic graph depicting hypothesized mediation pathway.

**Notes:** The hypothesized pathway for this study has two exposures: 1) implementation of the Patient-Driven Payment Model (PDPM) and 2) the onset of the COVID-19 pandemic, the mediator of therapy volume declines, confounders, and outcomes of successful community discharge and 30-day hospital readmissions. The indirect effect is the association between PDPM implementation and COVID-19 onset and outcomes that is mediated through the declines in therapy volume.


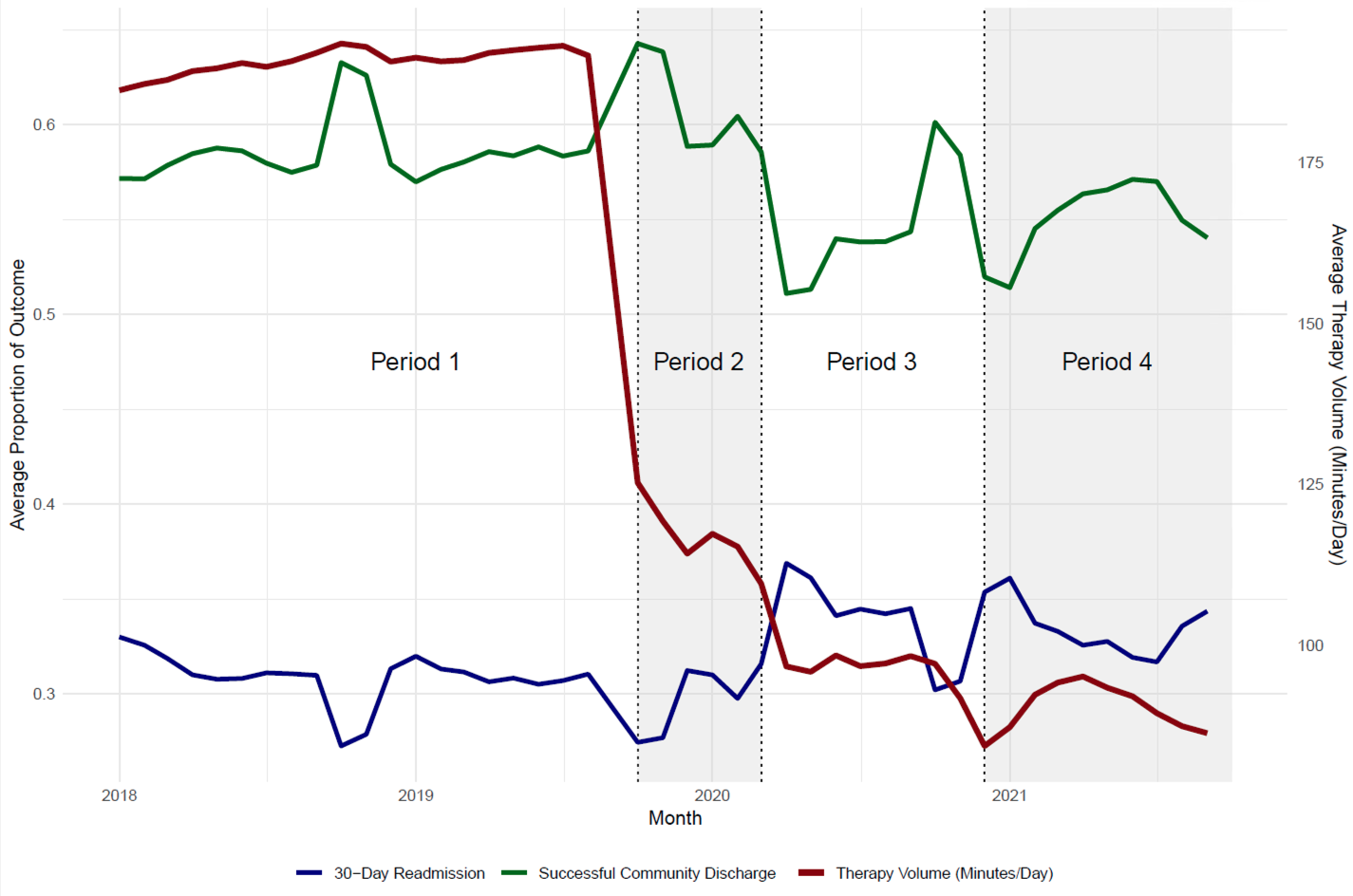


**Appendix Figure 3.** Unadjusted average therapy volumes and patient outcomes over time among 3,534,928 skilled nursing facility (SNF) stays for sensitivity analysis using multiple COVID-19 pandemic time periods.

**Notes:** Green and blue lines reflect monthly average proportion of SNF stays with successful community discharge and 30-day readmissions by time period, respectively. The red line shows the monthly average therapy volume, or total minutes of physical, occupational, and speech therapy per day of therapy during the SNF stay. Period 1: Before Patient-Driven Payment Model (PDPM) implementation (January 2018 through September 2019); Period 2: After PDPM Implementation, Before COVID-19 onset (October 2019 through February 2020); Period 3: Early COVID-19 (March 2020 through November 202), Period 4: Late COVID-19 (December 2020 through September 2021).

| **Appendix Table 2. Descriptive Statistics for Post-Acute Skilled Nursing Facility Stays by Study Time Period** | | | |
| --- | --- | --- | --- |
| **Patient Outcomes** | **Period 1**: Before PDPM Implementation  (N=1,995,196) | **Period 2:** After PDPM, Before COVID-19  (N=416,659) | **Period 3:** During COVID-19  (N=1,123,073) |
| Successful Discharge to the Community (No.,%) | 1166298 (58.5%) | 255452 (61.3%) | 618145 (55.0%) |
| 30-day hospital readmission (No., %) | 617101 (30.9%) | 122484 (29.4%) | 376652 (33.5%) |
| **Mediator** |  |  |  |
| Total Minutes of Therapy per Day, Mean (SD) | 122.20 (29.87) | 96.47 (39.97) | 87.68 (40.61) |
| **SNF Stay Characteristics** |  |  |  |
| Length of Stay in Days (Mean, SD) | 24.82 (18.95) | 25.89 (19.56) | 26.98 (20.66) |
| January Admission (No., %) | 212130 (10.6%) | 79974(19.2%) | 63837(5.7%) |
| February Admission (No., %) | 207092 (10.4%) | 69886(16.8%) | 59827 (5.3%) |
| March Admission (No., %) | 221153 (11.1%) | 0 (0%) | 125289 (11.1%) |
| April Admission (No., %) | 208267 (10.4%) | 0 (0%) | 105796 (9.4%) |
| May Admission (No., %) | 209013 (10.5%) | 0 (0%) | 114327 (10.2%) |
| June Admission (No., %) | 194459 (9.7%) | 0 (0%) | 125914 (11.2%) |
| July Admission (No., %) | 196627 (9.8%) | 0 (0%) | 128087 (11.4%) |
| August Admission (No., %) | 187068 (9.4%) | 0 (0%) | 124205 (11.1%) |
| September Admission (No., %) | 90842 (4.6%) | 0 (0%) | 123711 (11.0%) |
| October Admission (No., %) | 90770 (4.5%) | 90025 (21.6%) | 51527 (4.6%) |
| November Admission (No., %) | 86901 (4.4%) | 84440 (20.3%) | 45480 (4.0%) |
| December Admission (No., %) | 92098 (4.6%) | 92567 (22.2%) | 55720 (5.0%) |
| **Patient Demographics** |  |  |  |
| Age in Years (Mean, SD) | 78.28 (10.97) | 78.78 (10.66) | 78.65 (10.59) |
| Unknown Race (No., %) | 12770 (0.6%) | 2960 (0.7%) | 8941 (0.8%) |
| White (No., %) | 1684863 (84.4%) | 353574 (84.8%) | 946923 (84.3%) |
| Black (No., %) | 208949 (10.5%) | 41168 (9.9%) | 118380 (10.5%) |
| Other (No., %) | 21544 (1.1%) | 4918 (1.2%) | 12442 (1.1%) |
| Asian (No., %) | 26847 (1.3%) | 5764 (1.4%) | 14155 (1.3%) |
| Hispanic (No., %) | 30704 (1.5%) | 6256 (1.5%) | 16308 (1.5%) |
| North American Native (No., %) | 10743 (0.5%) | 2252 (0.5%) | 6571 (0.6%) |
| Female (No., %) | 1198981 (60.1%) | 254181 (61.0%) | 668463 (59.5%) |
| Dually Medicare-Medicaid Eligible (No., %) | 540944 (27.1%) | 108516 (26.0%) | 324029 (28.8%) |
| Married (No., %) | 669971 (33.6%) | 139353 (33.4%) | 355997 (31.7%) |
| Needs Interpreter (No., %) | 49703 (2.5%) | 10413 (2.5%) | 26242 (2.3%) |
| Patient Zip Code: Urban (No., %) | 1607260 (80.5%) | 337551 (81.0%) | 917329 (81.6%) |
| Patient Zip Code: Large Rural (No., %) | 220791 (11.1%) | 45037 (10.8%) | 117561 (10.5%) |
| Patient Zip Code: Small Rural (No., %) | 102324 (5.1%) | 20750 (5.0%) | 54188 (4.8%) |
| Patient Zip Code: Isolated Small Rural (No., %) | 66045 (3.3%) | 13554 (3.3%) | 34642 (3.1%) |
| **Inpatient Hospital Stay Characteristics** |  |  |  |
| Intensive Care Unit Stay (No., %) | 629247 (31.5%) | 132741 (31.8%) | 351792 (31.3%) |
| Surgical Procedure (No., %) | 1189899 (59.6%) | 249581 (59.9%) | 682997 (60.8%) |
| **Patient Clinical Characteristics** |  |  |  |
| Elixhauser Comorbidity Index (Mean, SD) | 11.88 (9.28) | 12.10 (9.31) | 12.62 (9.29) |
| ADL Scale at Admission (Mean, SD) | 16.97 (4.43) | 16.78 (4.49) | 17.36 (4.61) |
| Moderate to Severe Vision Impairment (No., %) | 92061 (4.6%) | 19289 (4.6%) | 55785 (5.0%) |
| Pain Affects Sleep or Activity (No., %) | 313957 (15.7%) | 70770 (17.0%) | 182512 (16.2%) |
| One or More Falls in Last 6 Months (No., %) | 862032 (43.2%) | 190511 (45.7%) | 526854 (46.9%) |
| Agitated or Reactive Behaviors: None (No., %) | 1856295 (93.0%) | 387381 (92.9%) | 1035062 (92.1%) |
| Agitated or Reactive Behaviors: Mild to Moderate (No., %) | 101309 (5.1%) | 21838 (5.2%) | 63678 (5.7%) |
| Agitated or Reactive Behaviors: Severe (No., %) | 32894 (1.6%) | 6552 (1.6%) | 21228 (1.9%) |
| Agitated or Reactive Behaviors: Very Severe (No., %) | 5922 (0.3%) | 1121 (0.3%) | 3752 (0.3%) |
| Delirium (No., %) | 125642 (6.3%) | 26971 (6.5%) | 86950 (7.7%) |
| Pressure Ulcer (No., %) | 285445 (14.3%) | 59078 (14.2%) | 192748 (17.2%) |
| Fully Continent (No., %) | 596475 (29.9%) | 120750 (29.0%) | 242404 (21.6%) |
| Alzheimer’s Disease or Dementia (No., %) | 736603 (36.9%) | 147284 (35.3%) | 454133 (40.4%) |
| Depression (No., %) | 977617 (49.0%) | 204445 (49.0%) | 597964 (53.2%) |
| **Special Treatments During SNF Stay** |  |  |  |
| Chemotherapy (No., %) | 13793 (0.7%) | 2811 (0.7%) | 6677 (0.6%) |
| Radiation (No., %) | 3995 (0.2%) | 700 (0.2%) | 1607 (0.1%) |
| Hemodialysis (No., %) | 97539 (4.9%) | 20078 (4.8%) | 57687 (5.1%) |
| Ventilator (No., %) | 4209 (0.2%) | 706 (0.2%) | 2813 (0.3%) |
| Transfusions (No., %) | 5554 (0.3%) | 1081 (0.3%) | 2557 (0.2%) |
| Hospice (No., %) | 723 (0.0%) | 118 (0.0%) | 592 (0.1%) |
| **Skilled Nursing Facility Characteristics** |  |  |  |
| Chain Facility (No., %) | 1172453 (58.7%) | 242201 (58.1%) | 655577 (58.3%) |
| Hospital Based (No., %) | 77052 (3.9%) | 15811 (3.8%) | 37736 (3.4%) |
| Average Patient Census (Mean, SD) | 107.41 (66.96) | 105.81 (67.37) | 93.63 (60.29) |
| For-profit ownership (No., %) | 1401857 (70.2%) | 288667 (69.2%) | 798173 (71.0%) |
| Government ownership (No., %) | 67562 (3.4%) | 13542 (3.2%) | 31952 (2.8%) |
| Not-for-profit ownership (No., %) | 527001 (26.4%) | 114683 (27.5%) | 293595 (26.1%) |
| Quality of Care Star Rating: 1 (No., %) | 53379 (2.7%) | 10646 (2.6%) | 26347 (2.3%) |
| Quality of Care Star Rating: 2 (No., %) | 160671 (8.0%) | 36435 (8.7%) | 88529 (7.9%) |
| Quality of Care Star Rating: 3 (No., %) | 294124 (14.7%) | 73714 (17.7%) | 193482 (17.2%) |
| Quality of Care Star Rating: 4 (No., %) | 450623 (22.6%) | 112708 (27.0%) | 305507 (27.2%) |
| Quality of Care Star Rating: 5 (No., %) | 1037623 (52.0%) | 183389 (44.0%) | 509855 (45.4%) |
| Percent Medicaid Patients (Mean, SD) | 48.80 (25.35) | 47.35 (26.03) | 48.59 (25.92) |
| Percent Medicare Patients (Mean, SD) | 22.60 (18.12) | 23.07 (18.88) | 22.52 (18.77) |
| Hours of nurse staffing per day (Mean, SD) | 3.69 (0.87) | 3.71 (0.88) | 3.71 (1.02) |
| Rural Location (No., %) | 301427 (15.1%) | 61206 (14.7%) | 155631 (13.8%) |
| Average COVID-19 Case Rates per 10,000 County Residents (Mean, SD) | 0.00 (0.00) | 0.23 (3.02) | 614.54 (471.65) |
| **State** |  |  |  |
| Alabama (No., %) | 31180 (1.6%) | 6692 (1.6%) | 15833 (1.4%) |
| Arkansas (No., %) | 17781 (0.9%) | 3788 (0.9%) | 9358 (0.8%) |
| Arizona (No., %) | 29701 (1.5%) | 6452 (1.5%) | 18491 (1.6%) |
| California (No., %) | 181692 (9.1%) | 39641 (9.5%) | 98919 (8.8%) |
| Colorado (No., %) | 19468 (1.0%) | 4955 (1.2%) | 13006 (1.2%) |
| Connecticut (No., %) | 36469 (1.8%) | 7511 (1.8%) | 20308 (1.8%) |
| Delaware (No., %) | 8854 (0.4%) | 1810 (0.4%) | 4968 (0.4%) |
| Florida (No., %) | 166579 (8.3%) | 33799 (8.1%) | 96959 (8.6%) |
| Georgia (No., %) | 40188 (2.0%) | 8295 (2.0%) | 22134 (2.0%) |
| Hawaii (No., %) | 3455 (0.2%) | 995 (0.2%) | 2871 (0.3%) |
| Iowa (No., %) | 20091 (1.0%) | 4220 (1.0%) | 11898 (1.1%) |
| Idaho (No., %) | 6034 (0.3%) | 1633 (0.4%) | 4198 (0.4%) |
| Illinois (No., %) | 110060 (5.5%) | 21331 (5.1%) | 59436 (5.3%) |
| Indiana (No., %) | 52510 (2.6%) | 12327 (3.0%) | 31027 (2.8%) |
| Kansas (No., %) | 20397 (1.0%) | 4769 (1.1%) | 13707 (1.2%) |
| Kentucky (No., %) | 32429 (1.6%) | 7020 (1.7%) | 17848 (1.6%) |
| Louisiana (No., %) | 17704 (0.9%) | 3606 (0.9%) | 9904 (0.9%) |
| Massachusetts (No., %) | 75195 (3.8%) | 15340 (3.7%) | 42566 (3.8%) |
| Maryland (No., %) | 59830 (3.0%) | 12332 (3.0%) | 36175 (3.2%) |
| Maine (No., %) | 9961 (0.5%) | 1725 (0.4%) | 4086 (0.4%) |
| Michigan (No., %) | 74583 (3.7%) | 14803 (3.6%) | 39221 (3.5%) |
| Minnesota (No., %) | 26524 (1.3%) | 6826 (1.6%) | 18873 (1.7%) |
| Missouri (No., %) | 39066 (2.0%) | 8247 (2.0%) | 22326 (2.0%) |
| Mississippi (No., %) | 18062 (0.9%) | 4449 (1.1%) | 11820 (1.1%) |
| Montana (No., %) | 5874 (0.3%) | 1008 (0.2%) | 2651 (0.2%) |
| North Carolina (No., %) | 67657 (3.4%) | 14650 (3.5%) | 37210 (3.3%) |
| North Dakota (No., %) | 3739 (0.2%) | 879 (0.2%) | 2565 (0.2%) |
| Nebraska (No., %) | 13402 (0.7%) | 2921 (0.7%) | 8614 (0.8%) |
| New Hampshire (No., %) | 10506 (0.5%) | 1991 (0.5%) | 4732 (0.4%) |
| New Jersey (No., %) | 94888 (4.8%) | 18910 (4.5%) | 53529 (4.8%) |
| New Mexico (No., %) | 7576 (0.4%) | 1700 (0.4%) | 4566 (0.4%) |
| Nevada (No., %) | 9377 (0.5%) | 1999 (0.5%) | 5963 (0.5%) |
| New York (No., %) | 120783 (6.1%) | 26196 (6.3%) | 76443 (6.8%) |
| Ohio (No., %) | 89380 (4.5%) | 18637 (4.5%) | 51093 (4.5%) |
| Oklahoma (No., %) | 23789 (1.2%) | 4787 (1.1%) | 13261 (1.2%) |
| Oregon (No., %) | 17087 (0.9%) | 3646 (0.9%) | 8542 (0.8%) |
| Pennsylvania (No., %) | 85945 (4.3%) | 17952 (4.3%) | 51671 (4.6%) |
| Rhode Island (No., %) | 9098 (0.5%) | 1965 (0.5%) | 4889 (0.4%) |
| South Carolina (No., %) | 30467 (1.5%) | 6442 (1.5%) | 17968 (1.6%) |
| South Dakota (No., %) | 4323 (0.2%) | 908 (0.2%) | 2615 (0.2%) |
| Tennessee (No., %) | 47574 (2.4%) | 11256 (2.7%) | 29127 (2.6%) |
| Texas (No., %) | 94057 (4.7%) | 13733 (3.3%) | 31549 (2.8%) |
| Utah (No., %) | 11952 (0.6%) | 2755 (0.7%) | 6685 (0.6%) |
| Virginia (No., %) | 60057 (3.0%) | 12962 (3.1%) | 34373 (3.1%) |
| Vermont (No., %) | 5839 (0.3%) | 1140 (0.3%) | 2959 (0.3%) |
| Washington (No., %) | 37862 (1.9%) | 7862 (1.9%) | 18939 (1.7%) |
| Wisconsin (No., %) | 33499 (1.7%) | 6925 (1.7%) | 20322 (1.8%) |
| West Virginia (No., %) | 9951 (0.5%) | 2352 (0.6%) | 5608 (0.5%) |
| Wyoming (No., %) | 2701 (0.1%) | 517 (0.1%) | 1267 (0.1%) |
| Abbreviations: PDPM – Patient Driven Payment Model; ADL – Activities of Daily Living | | | |

| **Appendix Table 3.**  Adjusted effects of Sensitivity analysis examining Patient Driven Payment Model (PDPM) implementation, Early COVID-19 onset, and Late COVID-19 onset on patient outcomes compared to baseline period (January 2018 through September 2019) with mediation analysis estimates of indirect effects of declining therapy volumes for 3,534,928 skilled nursing facility stays from January 2018 through September 2021. | | | |
| --- | --- | --- | --- |
|  | Total Effects  (95% CI) | Direct Effects  (95% CI) | Indirect Effects  (95% CI) |
| **Percentage Point Change in Probability Following PDPM Implementation** | | | |
| Successful Community Discharge | 0.9 (0.7, 1.1) | 5.2 (5.0, 5.4) | -4.3 (-4.4, -4.2) |
| 30-Day Hospital Readmissions | -0.2 (-0.4, -0.03) | -2.9 (-3.1, -2.7) | 2.7 (2.6, 2.8) |
| **Percentage Point Change in Probability Following Early COVID-19 Onset** | | | |
| Successful Community Discharge | -0.5 (-0.7, -0.3) | 4.8 (4.6, 5.1) | -5.3 (-5.4, -5.3) |
| 30-Day Hospital Readmissions | 0.8 (0.6, 1.0) | -2.6 (-2.8, -2.4) | 3.4 (3.3, 3.4) |
| **Percentage Point Change in Probability Following Late COVID-19 Onset** | | | |
| Successful Community Discharge | -2.0 (-2.5, -1.6) | 3.0 (2.5, 3.5) | -5.0 (-5.2, -4.9) |
| 30-Day Hospital Readmissions | 1.9 (1.5, 2.4) | -1.2 (-1.7, -0.8) | 3.2 (3.1, 3.3) |
| **Source:** Authors’ analysis of 2018 – 2021 Medicare Provider Analysis and Review, Master Beneficiary Summary Files, Minimum Data Set 3.0, and publicly available files from the Centers for Medicare and Medicaid Services.  **Notes:** Effects are estimated from multivariable linear regression models with facility and calendar month fixed effects and adjusted for time-varying beneficiary and facility-level characteristics. Standard errors were clustered at the beneficiary and facility level to account for repeated measures. Total effects reflect estimated changes in each outcome after PDPM implementation (October 2019), early COVID-19 onset (March 2020), and late COVID-19 onset (December 2020) compared to baseline and accounting for declining therapy volumes. Direct effects are adjusted estimates of PDPM and COVID-19 onset in the absence of the mediator of declining therapy volumes. Indirect effects are results of the mediation analysis, estimating the indirect effects of changes in therapy volumes (i.e., minutes of therapy per day) on the total effects. | | | |

| **Appendix Table 4.**  Adjusted effects of stratified sensitivity analysis examining Patient Driven Payment Model (PDPM) implementation and COVID-19 onset on patient outcomes compared to baseline period (January 2018 through September 2019) with mediation analysis estimates of indirect effects of declining therapy volumes. | | | |
| --- | --- | --- | --- |
|  | Total Effects  (95% CI) | Direct Effects  (95% CI) | Indirect Effects  (95% CI) |
| ***Patients with diagnosis of dementia, N=1,337,141*** | | | |
| **Percentage Point Change in Probability Following PDPM Implementation** | | | |
| Successful Community Discharge | 1.1 (0.8, 1.4) | 5.7 (5.4, 6.0) | -4.6 (-4.7, -4.5) |
| 30-Day Hospital Readmissions | -0.5 (-0.7, -0.2) | -3.3 (-3.6, -3.0) | 2.8 (2.9, 2.9) |
| **Percentage Point Change in Probability Following COVID-19 Onset** | | | |
| Successful Community Discharge | -1.7 (-2.1, -1.4) | 3.9 (3.5, 4.2) | -5.6 (-5.7, -5.5) |
| 30-Day Hospital Readmissions | 1.9 (1.5, 2.1) | -1.7 (-2.0, -1.4) | 3.5 (3.4, 3.6) |
| ***Patients with no diagnosis of dementia, N=2,197,596*** | | | |
| **Percentage Point Change in Probability Following PDPM Implementation** | | | |
| Successful Community Discharge | 0.9 (0.7, 1.1) | 5.0 (4.7, 5.2) | -4.1 (-4.2, -4.0) |
| 30-Day Hospital Readmissions | -0.1 (-0.3, 0.1) | -2.7 (-2.9, -2.4) | 2.5 (2.5, 2.6) |
| **Percentage Point Change in Probability Following COVID-19 Onset** | | | |
| Successful Community Discharge | 3.0 (0.1, 0.5) | 5.3 (5.1, 5.6) | -5.0 (-5.1, -4.9) |
| 30-Day Hospital Readmissions | 1.6 (-0.1, 0.4) | -3.0 (-3.2, -2.8) | 3.2 (3.1, 3.3) |
| **Source:** Authors’ analysis of 2018 – 2021 Medicare Provider Analysis and Review, Master Beneficiary Summary Files, Minimum Data Set 3.0, and publicly available files from the Centers for Medicare and Medicaid Services.  **Notes:** Effects are estimated from multivariable linear regression models with facility and calendar month fixed effects and adjusted for time-varying beneficiary and facility-level characteristics. Standard errors were clustered at the beneficiary and facility level to account for repeated measures. Total effects reflect estimated changes in each outcome after PDPM implementation (October 2019), and COVID-19 onset (March 2020) compared to baseline and accounting for declining therapy volumes. Direct effects are adjusted estimates of PDPM and COVID-19 onset in the absence of the mediator of declining therapy volumes. Indirect effects are results of the mediation analysis, estimating the indirect effects of changes in therapy volumes (i.e., minutes of therapy per day) on the total effects. | | | |

| **Appendix Table 5.**  Adjusted effects of stratified sensitivity analysis examining Patient Driven Payment Model (PDPM) implementation and COVID-19 onset on patient outcomes compared to baseline period (January 2018 through September 2019) with mediation analysis estimates of indirect effects of declining therapy volumes. | | | |
| --- | --- | --- | --- |
|  | Total Effects  (95% CI) | Direct Effects  (95% CI) | Indirect Effects  (95% CI) |
| ***Low function: Patients in lowest quartile of functional independence at admission, N=902,263*** | | | |
| **Percentage Point Change in Probability Following PDPM Implementation** | | | |
| Successful Community Discharge | 1.8 (1.4, 2.2) | 5.5 (5.1, 5.9) | -3.7 (-3.8, -3.6) |
| 30-Day Hospital Readmissions | -0.9 (-1.3, -0.5) | -3.0 (-3.4, -2.6) | 2.1 (2.0, 2.2) |
| **Percentage Point Change in Probability Following COVID-19 Onset** | | | |
| Successful Community Discharge | -0.4 (-0.7, -0.02) | 3.9 (4.5, 4.3) | -4.3 (-4.4, -4.1) |
| 30-Day Hospital Readmissions | 0.8 (0.5, 1.2) | -1.6 (-2.0, -1.2) | 2.4 (2.3, 2.5) |
| ***Moderate function: Patients in middle two quartiles of functional independence at admission, N=1,780,552*** | | | |
| **Percentage Point Change in Probability Following PDPM Implementation** | | | |
| Successful Community Discharge | 1.3 (1.0 1.5) | 5.9 (5.6, 6.1) | -4.6 (-4.7, -4.5) |
| 30-Day Hospital Readmissions | -0.4 (-0.7, -0.2) | -3.3 (-3.6, -3.1) | 2.9 (2.8, 3.0) |
| **Percentage Point Change in Probability Following COVID-19 Onset** | | | |
| Successful Community Discharge | 0.2 (-0.1, 0.5) | 5.8 (5.5, 6.1) | -5.6 (-5.8, -5.5) |
| 30-Day Hospital Readmissions | 0.3 (0.04, 0.6) | -3.2 (-3.5, -3.0) | 3.5 (3.4, 3.6) |
| ***High function: Patients in highest quartile of functional independence at admission, N=851,922*** | | | |
| **Percentage Point Change in Probability Following PDPM Implementation** | | | |
| Successful Community Discharge | 0.5 (0.2, 0.8) | 3.9 (3.5, 4.2) | -3.3 (-3.5, -3.2) |
| 30-Day Hospital Readmissions | 0.1 (-0.2, 0.4) | -2.1 (-2.4, -1.8) | 2.2 (2.1, 2.3) |
| **Percentage Point Change in Probability Following COVID-19 Onset** | | | |
| Successful Community Discharge | 0.5 (0.2, 0.9) | 4.9 (4.5, 5.3) | -4.3 (-4.5, -4.2) |
| 30-Day Hospital Readmissions | 0.03 (-0.3, 0.3) | -2.9 (-3.1, -2.5) | 2.8 (2.7, 2.9) |
| **Source:** Authors’ analysis of 2018 – 2021 Medicare Provider Analysis and Review, Master Beneficiary Summary Files, Minimum Data Set 3.0, and publicly available files from the Centers for Medicare and Medicaid Services.  **Notes:** Quartiles of functional impairment were calculated using the 28-point scale of combined function on seven activities of daily living on the admission Minimum Data Set. Higher numbers indicate higher functional impairment. In our sample, the lowest quartile of function had scores of 20 or higher, the highest quartile of function had scores of 14 or lower, and scores from 15-19 were in the middle two quartiles.  Effects are estimated from multivariable linear regression models with facility and calendar month fixed effects and adjusted for time-varying beneficiary and facility-level characteristics. Standard errors were clustered at the beneficiary and facility level to account for repeated measures. Total effects reflect estimated changes in each outcome after PDPM implementation (October 2019), and COVID-19 onset (March 2020) compared to baseline and accounting for declining therapy volumes. Direct effects are adjusted estimates of PDPM and COVID-19 onset in the absence of the mediator of declining therapy volumes. Indirect effects are results of the mediation analysis, estimating the indirect effects of changes in therapy volumes (i.e., minutes of therapy per day) on the total effects. | | | |
